# Perspectives of Frontline Clinicians and End-Line Users on Smartphone-Based Photography for Assessing Traumatic Dental Injuries: Focus Group Interview Study and Thematic Analysis

**DOI:** 10.2196/82668

**Published:** 2026-01-26

**Authors:** Emily C Schultz, Boyen Huang, Margaret Shenouda, Mohamed Estai, Sarbin Ranjitkar, Jeffrey P Louie, Patimaporn Pungchanchaikul

**Affiliations:** 1 Department of Primary Dental Care University of Minnesota School of Dentistry Minneapolis, MN United States; 2 Department of Dental Education College of Allied Health and Nursing Minnesota State University Mankato, MN United States; 3 School of Human Sciences The University of Western Australia Perth Australia; 4 School of Dentistry College of Health Adelaide University Adelaide Australia; 5 Department of Pediatrics University of Minnesota Medical School Minneapolis, MN United States; 6 Faculty of Dentistry Khon Kaen University Khon Kaen Thailand

**Keywords:** acceptability, dental trauma, feasibility, image quality, mHealth, teledentistry, telediagnosis, usability, user perception

## Abstract

**Background:**

Mobile health (mHealth) is increasingly used in teledentistry for telediagnosis and other services; yet, the perceptions of frontline clinicians and end-line users regarding these technologies remain unexplored.

**Objective:**

This study examined the acceptability, feasibility, and usability of an mHealth model for telediagnosis from the perspectives of frontline clinicians and end-line users.

**Methods:**

A qualitative study using focus group interviews was conducted with 15 participants, including frontline clinicians and end-line users. Frontline clinicians captured dental images via a smartphone app, while end-line users assessed them through an mHealth platform. Interview transcriptions were thematically analyzed using consensus coding.

**Results:**

Thematic analysis identified 9 key themes: feasibility and perceived ease of use, perceived usefulness, compatibility, self-image and social influences, self-efficacy, voluntariness and behavior intention, anxiety, facilitating conditions, and attitudes toward a behavior. Participants considered smartphone-based photography acceptable and feasible for remote dental assessment. Facilitators and barriers to implementing the mHealth model were highlighted, and recommendations for improvements were proposed.

**Conclusions:**

Cyclical education and professional development are essential to enhancing user confidence and technology usability. Addressing patient and clinician resistance through targeted education, improved communication, and operational upgrades such as camera grids, system integration, and simplified login can support adoption. This study highlights mHealth’s potential in emergency dental assessment and screening, particularly for underserved populations, and underscores opportunities for interprofessional collaboration. Future research should explore broader clinical applications across oral health conditions.

## Introduction

Traumatic dental injuries (TDIs) are prevalent [1] and demand urgent attention when accompanied by pain and/or bleeding [2]. Most patients with TDI presenting in emergency departments need consultation and evaluation from dental professionals [2]. However, the limited on-call availability of dental professionals in emergency settings often leads to unnecessary patient transfers and increased health care costs [3], an issue that worsened as dental emergency services declined during the COVID-19 pandemic [4]. To address these challenges, technologies for virtual dental examinations have been developed and implemented [5].

Mobile health (mHealth) facilitators have incorporated mobile phones and wireless technological devices into health care practices, supporting the promotion and maintenance of health, enhancing preventive care, improving clinical decision-making and operational efficiency, and enabling remote communication and interaction. This approach has gained widespread adoption among health care professionals and caregivers [6]. Key benefits of mHealth include increased accessibility to health care services, the provision of anonymous consultation, and decreased reliance on travel and physical contact. These advantages were particularly valuable during the COVID-19 pandemic, when social distancing measures were necessary [7].

The concept of mHealth as a modality of teledentistry, or more specifically, m-oral health [8], includes teleconsultation for treatment planning and review [9], telediagnosis with virtual examination [9-14], telemonitoring using patient-generated health data [15-18], telesupport with interactive multimedia [19], and teleintervention to improve therapy adherence and complication management [20]. Prior mHealth studies have demonstrated adequate diagnostic performance for the remote assessment of TDIs [10], dental caries [11], and oral cancer [12] using smartphone-acquired photographs. In a survey conducted in Saudi Arabia, more than 60% of dentists have used mHealth technologies to capture and/or transmit clinical photographs, and the majority of them were confident in the diagnostic accuracy using this approach [21].

Our recent work described a workflow for telediagnosis of TDI, where trained clinicians and students used a smartphone camera and app to capture dental photographs, uploading them to secure cloud storage. Remote dentists and dental therapists then reviewed the images, recorded dental findings, and provided clinical recommendations through the same pathway in reverse. While the quantitative performance metrics of this mHealth practice were promising, areas for improvement, such as image quality and professional development on dental photography and remote assessment, were also indicated [10].

Within the Technology Acceptance Model (TAM), perceived ease of use and perceived usefulness are the determinants for an individual’s intention to use a new technology [22]. Based on different roles and concerns, Wallis et al [23] categorized mHealth users as patients, frontline clinicians (point-of-care clinicians), and end-line users (academic experts). Building on a similar classification, the users of our telediagnosis model comprised human participants (patients and nonpatient individuals who provided consent for dental photography), frontline clinicians (those who captured dental photos), and end-line users (those who remotely assessed dental photos) [10]. When searching literature with keywords of feasibility, acceptability, usability, or user perception, we found that only a few studies have reported frontline clinicians’ and end-line users’ perspectives on specific mHealth models, practices, or technologies for dental care or oral health promotion [13,15-18,20]. Among these, only 1 study focused on telediagnosis [13], but their diagnostic method was not photography-related. Of further note, an earlier mHealth study reported the perspectives of parents and caregivers on a photographic telediagnosis model for the detection of dental caries [14], but they did not investigate the perspectives of frontline clinicians and end-line users.

Despite evidence that smartphone-acquired photographs can achieve acceptable diagnostic performance for several oral conditions, few studies have examined the perspectives of the 2 professional user groups essential to photographic telediagnosis, frontline clinicians who capture images and end-line users who interpret them. Prior work has focused on patients, caregivers, or nonphotography telemethods, leaving clinician-centered implementation questions unanswered. This gap matters because diagnostic accuracy alone does not guarantee real-world adoption. Human factors such as privacy concerns, workflow fit, training needs, and system integration also directly influence acceptability and sustained use. By exploring these clinician perspectives, this study addresses practical barriers and facilitators that must be resolved to translate promising diagnostic performance into scalable, safe, and equitable teledentistry services. This study therefore aimed to explore frontline clinicians’ and end-line users’ perspectives on the feasibility, usability, and acceptability of smartphone-based photographic telediagnosis for TDI.

## Methods

### Study Design and Setting

This qualitative study was part of a larger project and was reviewed and approved by the University of Minnesota Institutional Review Board (Study ID: STUDY00014736). The study setting consisted of multiple sites located in Minnesota (United States) and Khon Kaen (Thailand), including the University of Minnesota clinical site, Khon Kaen University clinical sites, and Minnesota State Fair research facilities. The quantitative component of the larger project has been reported recently [10]. Following that, a qualitative study design grounded in the TAM [22] and interpretivism [24] was used to evaluate the perspectives of frontline clinicians (dental photographers) and end-line users (remote reviewers) on their use of the mHealth technology to assess TDI. Qualitative study data were collected in November and December 2023 and analyzed from January to August 2024. The conduction and presentation of this study adhered to the COREQ (Consolidated Criteria for Reporting Qualitative Research) guidelines [25].

**Figure 1:**
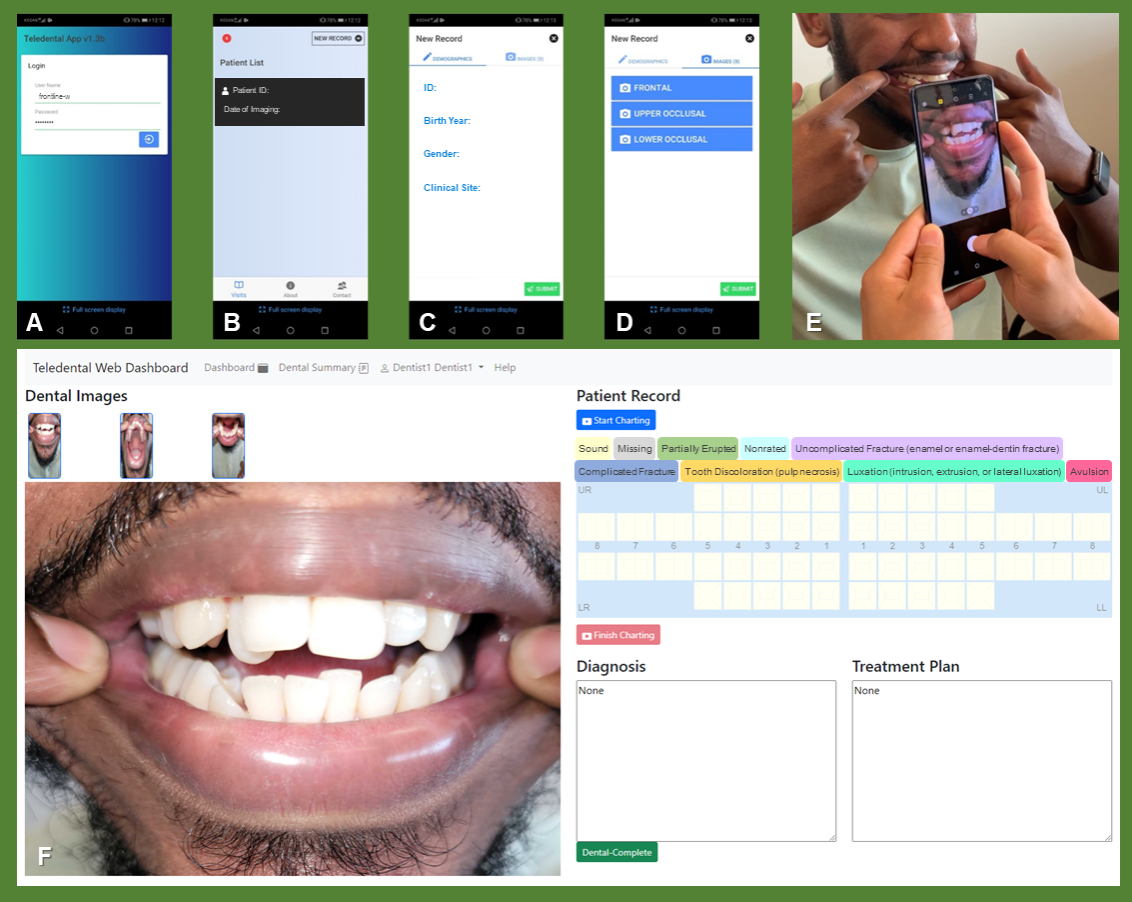
Workflow and interfaces of the mHealth app and platform used in this study for photographic telediagnosis of traumatic dental injuries.

This study built upon our quantitative work of TDI remote assessments using an image acquisition app “Teledental” (Commonwealth Scientific and Industrial Research Organisation [CSIRO]) and a web-based data management platform “Remote-I” (CSIRO; Figure 1). From August 2022 to July 2023, the photographs of teeth were captured by 11 trained frontline clinicians using the Teledental app, and then remotely reviewed by 5 licensed dental professionals (end-line users) using the Remote-I system [10].

In Figure 1, panels A-C show the “Teledental” app interface used by frontline clinicians to log in with individual passwords and enter patient data, including patient ID, birth year, sex, and clinical site. Panel D displays the app’s imaging screen, which prompts users to capture 3 standardized intraoral views: frontal, upper occlusal, and lower occlusal. Panel E depicts 2 frontline clinicians participating in one-on-one calibration after completing a training session designed to ensure consistent image quality and adherence to capture protocols. Panel F shows the “Remote-I” platform interface used by end-line users to access the patient record created during calibration; when the cursor hovers over a thumbnail, the corresponding dental image is enlarged for review. End-line users examined all 3 images and charted dental conditions using a color-coded odontogram that included diagnostic categories such as uncomplicated fractures, luxations, and partially erupted teeth. The clinicians provided consent for the use of their images and data in the publication.

### Basic Security Measures

All researchers, frontline clinicians, and end-line users adhered to institutional review board–approved procedures governing data access and privacy. To limit image persistence on devices, the Teledental app was configured to prevent local storage of captured images; images were transmitted directly to a US‑owned cloud service selected jointly by the research team and the vendor (CSIRO). Remote image review required authenticated access to the Remote‑I platform; the platform’s settings disallowed image downloads to end-line users’ workstations. Study smartphones were purchased new from the vendor (Samsung), configured with restricted user permissions, and connected only to password‑protected institutional Wi‑Fi (eg, eduroam or hospital internal networks); installation of additional apps was prohibited; devices were centrally stored and managed by a key researcher in Minnesota and a key researcher in Khon Kaen. Individual user accounts and passwords were required for both Teledental and Remote‑I and the passwords were changed periodically. Two‑factor authentication was not used by study participants during data collection. All study personnel completed training in privacy and data‑handling best practices, including HIPAA (Health Insurance Portability and Accountability Act) principles [26], and compliance with those requirements was monitored.

### Participants

All eligible frontline clinicians and end-line users who contributed to the preceding quantitative study were invited (consecutive sampling) and the full available cohort consented to participate, with the only exclusion being a frontline clinician who is a coauthor to avoid conflict of interest. The sample included a purposeful mix of roles and experience levels (dental students, general dentists, dental therapists, and dental specialists) across 2 countries. No participants were selected or excluded by demographic or professional criteria. To acknowledge potential recruitment influences, we report that University of Minnesota employees and student workers received modest incentives (US $40 and US $15, respectively) while participants based in Thailand did not. All participants were notified of the requirement to attend a 60-minute focus group interview through the Zoom app (Zoom Video Communications). Each focus group was composed of 1 to 6 participants according to their schedule availability. The participants based in the United States and Thailand were separated into different focus groups due to different time zones. The interviews were conducted in English by 2 US-based researchers together. All participants were capable of communicating in English during the Zoom interviews.

### Data Collection and Instrument

Two moderators, ECS (a female dental hygiene faculty member with a Master of Science degree) and MS (a female Honors college student with a Bachelor of Arts degree) conducted each focus group interview, while BH (a male senior dentistry faculty member and the principal investigator [PI] with a Doctor of Philosophy and Doctor of Dental Surgery degrees) observed all sessions. Prior to the first interview, the moderators underwent training to adopt a neutral interviewing stance, ensuring that participants’ perspectives were accurately captured. Since participants were recruited from frontline and end-line users of a previous study led by the observer, the moderators had a limited prior acquaintance with 3 participants. To maintain transparency, the moderators and observer introduced themselves and outlined the research objectives and content on the informed consent form and at the beginning of each interview. Before the interview with participants based in Thailand, the observer and moderators interacted with them in English for approximately 15 minutes via Zoom to confirm their proficiency and ensure effective communication. Only the participants and researchers were present during the interviews. Field notes were taken by the moderators during the sessions, and all interviews were audio-recorded and securely stored on a cloud server. After each focus group, participants were emailed the full study instrument and given 2 weeks to provide additional comments. To safeguard data quality and reduce risks of misinterpretation, a researcher (PP), a native Thai speaker fluent in English with graduate training in Britain, attended the Thai focus group to provide Zoom technical support and reviewed the transcripts from that session for accuracy. Although data saturation was defined as the point at which no new information emerged across 3 consecutive focus groups [27], we chose to interview all 15 participants in an effort to generate more evidence and decrease bias due to our small but very diverse sample size. Repeat interviews were not conducted.

The study instrument used for the interviews consisted of guided interview questions, exploring the perspectives of the frontline clinicians and end-line users. The interview questions were adapted from published resources [22,28-32] and were rephrased to reflect the context of this qualitative study and the mHealth technologies used. Subsequently, a multimember researcher panel with diverse expertise, including qualitative study design, medical informatics, clinical dentistry, dental public health, and special needs dentistry, reviewed and finalized the questions. The interview questions included in this study are presented in Table 1. The purpose of using this guided interview instrument for the interview was to gain a well-rounded perspective from the participants on their experience with the mHealth model and maintain consistency among all focus group interviews.

**Table 1:** Guided interview questions adapted from the Technology Acceptance Model.

Interview questions	References
Tell us about the feasibility of using the mHealth^a^ app or platform. What factors played a role?	[22,28]
How do you feel this mHealth app or platform can contribute to working with dental trauma cases? (In terms of efficiency, usefulness, etc)	[22,28]
How does this mHealth app or platform fit with the way dental trauma cases are examined and diagnosed?	[28,29]
How do you feel the use of the mHealth app or platform may change the way you and other people in your profession, specialty or field are viewed?	[28,29]
Did your previous experiences influence your use of the mHealth app or platform? Anything positive? Anything negative?	[28,30]
How willing are you to use the mHealth app or platform to examine and diagnose dental trauma cases in the future?	[28,29]
How likely are you to use an mHealth app or platform like the one we used in the future for dental trauma cases?	[28,31]
How comfortable or uncomfortable did you feel about using the technology for dental trauma cases? Only to frontline clinicians: How might you have observed either potential distress or comfort from the patients or fairgoers enrolled in this study?	[28,30]
What attitudes do you believe your superiors have around your use of the mHealth app or platform to examine and diagnose dental trauma cases?	[28,31]
What resources, knowledge, or assistance do you feel should be available when using the mHealth app or platform to examine and diagnose dental trauma cases?	[28,32]
What do you feel went well with the mHealth app or platform? What improvements do you feel could be made with the mHealth app or platform?	[28,31]

^a^mHealth: mobile health.

### Data Analysis

A consensus coding process was used to summarize and synthesize the qualitative data from the interview questions and determine the results [33,34]. Coders calibrated via Zoom before conducting any focus group interviews. This early calibration ensured alignment in coding practices and interpretation prior to engaging with the data, to maintain consistency across all transcripts. Memoing was used throughout the coding process to document coder reflections, emerging themes, and the rationale behind coding decisions. These memos supported transparency and helped track the coding development. The research team used a negotiated agreement strategy for intercoder reliability. Prior to data collection, coders collaboratively established a process for resolving discrepancies. When disagreements arose during coding, coders revisited the transcript, discussed their individual interpretations, and reached a consensus. The PI, who observed all focus group interviews, was also consulted to help resolve disagreements and ensure consistency. We used a hybrid coding approach that integrated both deductive and inductive strategies to analyze the focus group data. Initially, we developed a set of a priori codes grounded in our research questions and informed by the TAM [22], which provided a theoretical framework for examining participants’ responses. As coding progressed, we remained open to emergent patterns and concepts, allowing for the creation of new inductive codes that captured unanticipated insights arising directly from the data. This flexible approach enabled us to systematically explore expected themes while also incorporating novel findings that enriched our understanding of participants’ experiences [34,35]. After all interview sessions had taken place, a Zoom meeting among the research team was held to analyze and discuss the qualitative data, use the transcripts to identify common themes, and extract appropriate evidence, such as descriptive terms and quotes, from the participants’ responses. The transcripts and findings were not returned to participants for review.

### Ethical Considerations

This study was reviewed and approved by the University of Minnesota Institutional Review Board (Study ID: STUDY00014736). Written informed consent was obtained from all participants prior to data collection. All researchers and study personnel adhered to institutional review board–approved procedures governing data access and privacy. Compensation was provided to University of Minnesota employees and student workers, but not Thailand-based participants due to University out of country payment restrictions.

## Results

### Overview

A total of 7 interview sessions were conducted. Among the 15 study participants, 10 were frontline clinicians who took the dental photographs and 5 were end-line users who interpreted the dental photographs from a distance. The 10 frontline clinicians included 5 general dentists, 1 pediatric dental specialist, 1 dental therapist, and 3 dental students. The 5 end-line users consisted of 1 pediatric dental specialist, 2 general dentists, and 2 dental therapists. The 6 dentists who acted as frontline clinicians and took dental photographs were all based in Khon Kaen (Thailand), while the other 4 frontline clinicians and the 5 end-line users were based in Minnesota (United States). Table 2 lists the focus groups, roles, professions, sex, and countries of the participants included in this qualitative study.

Data saturation was achieved within the 7 interviews as no new codes or themes emerged after the first 4 interviews. Thematic analysis of frontline clinicians’ and end-line users’ structured interviews revealed 9 main themes: feasibility and perceived ease of use, perceived usefulness, compatibility, self-image and social influences, self-efficacy, voluntariness and behavior intention, anxiety, facilitating conditions, and attitudes toward a behavior (Table 3).

**Table 2:** Focus groups, roles, professions, sex, countries, and years in practice of the participants (9 in the United States and 6 in Thailand); years in practice ranged from 0 to 20 years. All consented to publication.

Focus group and role		Profession	Sex	Country	Years in practice
1					
	End-line user	Dental therapist	Male	United States	10
	End-line user	General dentist	Male	United States	15
2					
	End-line user	Dental therapist	Female	United States	2
3					
	Frontline clinician	Dental student	Male	United States	0
4					
	Frontline clinician	Dental therapist	Female	United States	12
	Frontline clinician	Dental student	Male	United States	0
5					
	End-line user	General dentist	Male	United States	3
	End-line user	Dental specialist	Male	United States	20
6					
	Frontline clinician	Dental student	Female	United States	0
7					
	Frontline clinician	General dentist	Female	Thailand	5
	Frontline clinician	General dentist	Female	Thailand	6
	Frontline clinician	General dentist	Female	Thailand	12
	Frontline clinician	General dentist	Male	Thailand	5
	Frontline clinician	General dentist	Male	Thailand	8
	Frontline clinician	Dental specialist	Male	Thailand	15

**Table 3:** Subthemes (code words), definitions, and representative transcript excerpts that contributed to theme construction.

Main theme		Definition	Subtheme	Example from transcripts
Feasibility and perceived ease of use		Statements that describe the feasibility and ease of use of the mHealth^a^	Consistency Login Template	“I think it was fairly easy for me just because I was comfortable using intraoral cameras before. I think it’s something that would be kind of intuitive to a new user. It wasn’t that difficult.” “I think the only negative thing, which we kind of figured out, was if the app was left unutilized for a certain amount of time, then it would time out and you’d have to sign back in.” “I want a guideline with the application while using. Kind of like a template to make sure pictures are being taken at correct angles.”
Perceived usefulness		Comments relating to the usefulness of mHealth	Access Timely Useful	“I think it’s beneficial because, in terms of increasing access, people might not always have access to dental services right away following trauma.” “mHealth could help get the treatment done faster if you could get diagnosis back quicker and in turn get patients treatment faster.” “Specifically speaking about usability, it was really user-friendly in terms of clicking on patient, reviewing what is in odontogram and chart.”
Compatibility		Statements referring to the familiarity and integration of mHealth technologies	Technology familiarity	“I think with our generation in this day and age it is easier to use apps and the app wasn’t that complicated at all, it was kind of like straightforward.”
Self-image and social influences		Comments that describe the social perception of mHealth use	Acceptance Resistance	“I feel like this would be viewed in a positive manner, in that we are taking extra steps to expand access to care through teledentistry and through this mHealth method of like this is easily accessible for patients.” “Some people might say well that's not truly seeing the patient or diagnosing the issue if you're not seeing them in-person since it's kind of a new area of dentistry, but I don’t think I'd have any issue with performing teledentistry.”
Self-efficacy		Statements that describe the user’s beliefs in themselves to use mHealth	Confidence	“I was very comfortable with how to utilize the technology and then since I’ve used my phone a lot and taken a lot of images before, it was pretty streamline for me to be able to use.”
Voluntariness and behavior intention		Comments referring to the user’s intention to adopt or integrate mHealth into practice	Reluctance Intention	“For me, I don’t feel like it adds a lot to either the patient experience or patient care at the moment.” “I think I would definitely be willing to use it, like I was saying, like for screening patients, I think it’s a very valuable asset to see how acute or how soon they would need to get in.”
Anxiety		Statements that describe the user’s comfort level related to the use of mHealth	Comfortable	“I think it was very easy for me. I think it was something that I felt very comfortable being able to use, taking the images, making sure the images came out well. I felt very comfortable doing that. I know it may be more challenging for people who are not as familiar with being able to use that technology.”
Facilitating conditions	Statements that describe user’s view on what conditions are needed to implement mHealth		Resources More practice	“I think the research team did a good job training us through this, the app, and showing us how to use it, and they even did a one-on-one calibration when we got the phones from them.” “I think if we did a few practice sessions it would help us a lot to be able to emulate what type of images we wanted to take and how we wanted them to look like. So providing some model images of what the appropriate images would look like, I think that would be beneficial.”
Attitudes toward a behavior	Statements that describe a user’s feelings about the use of mHealth		Positive attitudes Improvements	“I was excited when I was using it. I thought it’s something that I can definitely make a difference.” “Technology that would streamline the photo taking process, instead of needing to click. Something that would eliminate the need to save or change to the next photo manually. Also not having to login every time, save login for quicker future login.”

^a^mHealth: mobile health.

### Feasibility and Perceived Ease of Use

As a whole, the frontline clinicians based in Thailand and the United States found the mHealth app feasible and straightforward to use, stating the app itself was self-explanatory, simple, and easy to use. The frontline clinicians at the Minnesota State Fair (United States) used the technology more often and more consistently, while the frontline clinicians at clinical sites in Minnesota (United States) and Khon Kaen (Thailand) used the technology more sporadically. With this being said, frontline clinicians who used it more consistently found it easier to use than those who used it sporadically as they were more familiar with the technology.

Almost all frontline clinicians felt that a more streamlined login process is necessary to improve mHealth app use. They criticized the need to log in each use with a username and password, as it could be time-consuming. Frontline clinicians in the United States suggested some options for streamlining the login process, including facial recognition for login, the ability to stay logged in and/or the ability to save username and password on the login page. Both frontline clinicians in Thailand and the United States mentioned lighting and picture scale as potential barriers. Both parties suggested some type of template or guide to help with image flow, image angles, and image scale and believe this would make images more consistent. A frontline clinician also mentioned that at times he would have trouble focusing the image,

For some reason, when I was taking photos, I felt like I couldn’t focus on what I wanted to focus on. Sometimes, I would take multiple pictures of the same thing and try to get a really good picture, but I couldn’t get a clear image of things.

End-line users had similar feelings to the frontline clinicians as far as the feasibility of the mHealth data management platform. Generally speaking, end-line users mentioned the platform itself was feasible, simple to use, user-friendly and straightforward. Regarding the mHealth platform, a dentist end-line user stated, “I think it’s something that would be kind of intuitive to a new user.”

A dental specialist and a general dentist mentioned that if a charting error was made, there was not a simple way to correct the mistake. They found the easiest way to correct it was to exit out of the platform and start over. They mentioned the importance of fixing this, because they believe teledentistry should be fairly efficient due to the volume one may be reviewing. A dental therapist end-line user stated that the most difficult thing was that the platform could be slow at times. Despite the hardships, overall end-line users found the learning curve to be manageable.

### Perceived Usefulness

As a whole, frontline clinicians perceived the mHealth app to be useful. Several frontline clinicians believed that the mHealth app was beneficial, particularly for increasing access to dental care and reducing the burden on emergency services. Frontline clinicians based in the United States commented, “People might not have access to dental services right away following trauma.” They also stated, “I believe this app is very useful and beneficial, especially like when kids get trauma and they don’t have a dental home, it gives them quick access to care.” Frontline clinicians in Thailand also thought that the app would be a game changer for teledentistry. Specifically, they thought the mHealth app was great for those in remote areas where they do not have a dental specialist.

Frontline clinicians in both countries did suggest they think the app would be more useful if it could include more information, such as patient age, radiographs, and better image quality. They also felt some limitations with its current scope of only TDI and suggested widening it to a broader and more general dentistry scope.

End-line users deemed the mHealth data management platform useful for teledentistry, especially for initial assessments. They mentioned its potential for timely diagnosis and usefulness when there is not a dental provider in the nearby area.

Similar to the frontline clinicians, end-line users also felt having more patient information, such as pain, chief complaint, chart notes, etc, would be beneficial and useful. They also mentioned that suboptimal image quality of some dental photos may hinder the diagnosis of minor trauma and recommended that this be addressed for future use.

### Compatibility

Both frontline clinicians and end-line users felt that the mHealth app and platform were compatible with the way existing dental cases and TDI cases are treated. Meaning both parties felt the technology was not too out of the realm and could be useful for handling trauma cases. A frontline clinician and an end-line user eluded to the fact that “as a society we use technology every day, and some of the younger generations rely on technology so we are already familiar with it.” They stated that most people are using smartphones every day, so there likely would not be a learning curve for widespread TDI-based teledentistry. Future improvements for the mHealth app and platform, as recommended by the interviewees, included fine-tuning of the odontogram and patient chart displayed on the platform, and integration of the mHealth model with the practice management software available in the market.

### Self-Image and Social Influences

The frontline clinicians expressed that using the mHealth app did not significantly alter their self-image or how they were perceived by others. They felt the app was generally viewed positively as a helpful tool in the dental care process. A frontline clinician in Thailand stated, “there have been no bad attitudes from superiors when using the application.”

Although frontline clinicians generally had a positive perception of using an mHealth app, some concerns were raised about patients questioning the provider’s competence if they relied heavily on the app for diagnosis. One participant mentioned, “If the parent sees a provider using the application on their child they may question their intelligence as to why they are not confident in diagnosing on their own.” They also alluded that older generations may potentially have similar concerns.

Four out of 5 end-line users felt that using a teledentistry platform, such as mHealth, would have a positive impact on their professional images. They mentioned it would showcase their adaptability to using new technologies. The other end-line user, a general dentist, thought that professionals in the field would not view him or his work any differently whether he used the technology or not.

The only concern mentioned was initially, there may be some pushback, as health professionals have seen this when implementing other new technologies until the entire team is familiar and comfortable with it.

### Self-Efficacy

Frontline clinicians felt confident using the mHealth app after the initial training, citing their familiarity with smartphone technology as a contributing factor. One frontline clinician stated, “Once we had the training, I was very comfortable with how to utilize the technology and then since I’ve used my phone a lot and taken a lot of images before, it was pretty streamline for me to be able to use.” Another made a similar comment,

I felt pretty comfortable. I felt familiar with using a lot of apps and utilizing that app wasn’t very challenging, and then taking the images were pretty easy.

All end-line users also felt confident in their ability to use the mHealth platform effectively after receiving the initial training. End-line users attributed their confidence to the training session and prior experiences with other electronic charting and dental record systems. There were no barriers or negative experiences in regards to self-efficacy reported by end-line users.

### Voluntariness and Behavioral Intention

Frontline clinicians indicated a willingness to use the app voluntarily, recognizing its potential benefits in improving access to care and streamlining the diagnostic process. One participant said, “I think it’s beneficial because, in terms of increasing access, people might not always have access to dental services right away following trauma.”

They also suggested the app could be more widely adopted if it included additional features and addressed current limitations, such as the need for frequent logins and better image quality mentioned earlier.

There was a strong willingness among end-line users to adopt the mHealth platform for future use, especially for screening and remote consultations. Most of them saw it as a great triage tool to get their patients to the right place in a timely manner. End-line users saw it as a valuable tool for patient care and were open to integrating it into their daily practices, despite some initial hesitations. Per one end-line user, “I would definitely be willing to use it, I think it’s a very valuable asset.” Another end-line user stated, “I’d say I’m very likely to use it in the future.”

One end-line user, the dental specialist, was concerned with how the app would integrate into other dental practice software in an attempt to have more patient information available for the reviewer. Another end-line user, a general dentist, thought it would be beneficial to be able to communicate with the patient through the app, whether that be through voice, FaceTime (Apple Inc), or text messaging.

### Anxiety

The frontline clinicians overall seemed comfortable with using and capturing photos on the smartphones, as they use them regularly. Initial anxiety among frontline clinicians was noted due to the unfamiliarity with using the specific mobile app for dental assessments. Their anxiety lessened over time as they became more familiar with the process. Those frontline clinicians witnessed a wide range of anxiety among patients and state fairgoers who were getting their dental photos taken. The most anxiety from patients was reported in a Minnesota community clinical setting. Frontline clinicians attributed this mostly to privacy issues, stating that some of the patients are potentially illegal immigrants, and became hesitant once they found out their picture would be acquired. However, anxiety decreased among some patients once they fully understood the study purpose and applications as well as the photos taken were limited to their mouths. Minnesota state fairgoers were less anxious, as they had agreed to come to the research facilities and learn more about the study, and willingly came in to learn about the study, versus being at the dentist for an oral health issue. The frontline clinicians in Thailand did not notice much anxiety from their patients. They credited this to previous use of intraoral cameras during dental examinations and the fact that participants had previously gone through and provided an informed consent.

When it came to using the mHealth platform, end-line users were overall comfortable and felt the dental charting they did was straightforward. They did report anxiety when it came to system charting glitches, such as forgetting which teeth they had already marked as sound, and the accuracy of their diagnoses when the quality of some images was less than ideal.

### Facilitating Conditions

Frontline clinicians appreciated the training sessions and written materials provided, which helped them navigate the app effectively. One frontline clinician in the United States stated,

I think the research team did a good job training us through this, the app, and showing us how to use it, and they even did a one-on-one calibration when we got the phones from them.

The same interviewee thought that a couple of practice sessions and being provided with model images would have helped them have a better understanding and emulate the photo expectations. The frontline clinicians based in Thailand thought a video tutorial would have helped them better understand what angles they should take photos from. They found the most difficult part was needing to log in to the app and connect to the Wi-Fi every time they went to use the app.

When it came to resources, knowledge, or assistance, end-line users emphasized the importance of adequate training and support to maximize the platform’s effectiveness. They felt the training they received from the research team was adequate, and liked the guidelines so everyone was working off the same standards. However, an end-line user suggested that going through a couple of cases together would have been even more of help.

As stated earlier in this article, end-line users felt that including more patient information, or integrating with patients’ dental practice software to have access to patient dental history would make the diagnosis process easier.

### Attitudes Toward a Behavior

Overall, the frontline clinicians had a positive attitude toward using the mHealth app, as it was straightforward to use and they recognized its potential to enhance dental care delivery. They thought that if this dental model were integrated more widely, it could make a significant difference, especially in areas with a shortage of dental health professionals. The mHealth model was seen as a step forward in leveraging technology to improve health care access and efficiency.

Frontline clinicians thought the experience could be even more positive by suggesting things, such as streamlining the technology by automating the photo-taking process to save and switch to the next photo without manual intervention, and user interface enhancements, such as making the app more child-friendly with colors or cartoons.

Overall, the end-line users’ attitudes toward using the mHealth platform were positive. End-line users acknowledged its potential to streamline workflows and improve patient outcomes. They appreciated the convenience and accessibility it offered. Specifically, they noted the value of having standardized views for each patient and appreciated the ability to zoom in on the photos and the ability to chart multiple conditions on the tooth.

Despite the positive attitudes toward the platform and use, end-line users recognized some areas of improvement and future development. One suggestion in an attempt to standardize the diagnosis was to integrate the trauma guidelines into the platform for quick reference. Another end-line user suggested making the reviewing platform into an app that could be easily accessed with a smartphone to improve convenience during emergency situations.

## Discussion

### Principal Findings

This study revealed participant perspectives toward the mHealth app and platform. Participants highlighted improved and faster diagnosis, underscoring perceived usefulness, and generally found the system easy to use despite login and photo clarity challenges. The app and platform seemed compatible with current dental practices. Most users responded positively, though some worried patients might perceive overreliance on technology. Training increased confidence and reduced anxiety, leading to stronger self-efficacy. Some were ready to keep using the app, while others wanted improvements first, showing mixed intentions. Overall, users expressed positive attitudes toward its role in TDI assessment.

The 9 themes illustrate how TAM constructs operate in practice. Perceived usefulness related to faster diagnosis and improved access, while ease of use was reflected in straightforward operation. Compatibility was evident through integration with existing practices, and self‑efficacy was reinforced by training and calibration. Social influences appeared in concerns about patient perceptions and peer resistance, while voluntariness and behavioral intention were expressed in willingness or reluctance to continue use. Anxiety, facilitating conditions, and attitudes toward use further shaped adoption, underscoring the interplay between technical usability and human factors.

When implementing teledentistry in clinical settings, its impacts on oral health outcomes [35], patient satisfaction [36], and cost-effectiveness [35] are commonly taken into account, and these effects have been regularly reported in literature [16,17,20,35,36]. Despite our belief in patient-centered care [37,38], health care workers’ roles are central to mHealth adoption. In this study, smartphone-based photography was regarded as feasible, acceptable, and usable for remote TDI assessment. The results suggested potential applications in the initial assessment of emergency cases (such as trauma) and in triage or screening contexts (such as dental caries), warranting empirical testing in settings serving underserved populations and within interprofessional emergency workflows [2]. A prior caregiver study using the same mHealth app for dental screening and caries assessment in children also supported its feasibility [14], but direct comparisons are limited and require confirmatory research. Several other studies have also highlighted the relevance of including frontline and end-line users’ perspectives in the determination of feasibility, acceptability, and usability of mHealth technologies for dental care and oral health promotion [13,15-18,20]. Those studies reported benefits including diagnostic accuracy [13], communication [17], patient-provider relationship [17], efficiency [18], and medication adherence [20]. Compellingly, some users of a non–photography-related mHealth app recommended adding a photo-capture-and-forward function to it [15], which happens to be a key characteristic of our mHealth model. This could also imply the viability of smartphone-based photography in the mHealth modality of teledentistry.

Training and calibration were key facilitators for the use of this mHealth model, increasing comfort and confidence among both frontline clinicians and end-line users. Literature suggested that adequate training decreases uncertainty, strengthens self-efficacy, and enhances mHealth usability [10,15,39,40]. A recent scoping review has found approximately 20 teledentistry educational programs [41]. Building on this evidence, curricula should incorporate topics such as virtual communication and assessment, technology troubleshooting, billing, and ethics, to help learners develop teledentistry competencies. These could be complemented by simulated training and clinical practice [42]. Because familiarity enhances usability, periodic and continuous training is recommended to refresh skills and sustain competence.

Patient and clinician perceptions may pose barriers. Some older patients might view smartphone use during diagnosis as a sign of incompetence, while younger or tech-savvy patients were more accepting [43]. Education to improve digital literacy and highlight benefits can support adoption. Resistance from colleagues may also hinder uptake. Inclusive development, professional development, enhanced communication, and reduced operational burdens can facilitate acceptance [44].

This study is exploratory and focused on user perceptions rather than on product design or feature validation; nevertheless, participants also proposed technical suggestions. Consistent image quality is essential, and camera grids or templates could help. Integration of electronic patient records would strengthen utility, while governance and ethical frameworks addressing data linkage, artificial intelligence (AI), and security are needed [45,46]. Biometric or voice login could reduce screen contact while preserving security. Finally, a risk‑averse “ethical brake” approach—safeguards that trigger additional verification before data sharing or high‑stakes decisions—has been proposed to inform the design of login, data sharing, and reviewer feedback loops, helping minimize potential harms as mHealth and AI features are introduced [46].

To ensure trustworthiness, investigator and data source triangulation were used to test the validity [47]. The interview questions were created and reviewed by a multimember researcher panel. Two moderators and an observer were jointly involved in data collection and analysis. These approaches broadened the focus of this research and reduced researcher biases. Additionally, the data were collected from the participants of different roles (frontline clinicians and end-line users), qualifications (dental specialists, general dentists, dental therapists, and dental students), sexes (females and males), and locations (Thailand and the United States). This participant pool contributed to diverse perspectives and validation of data.

Using a focus group interview method presents certain limitations. Group composition was based on participants’ availability rather than self-selection, which may have influenced dynamics. As noted in prior literature, dominant voices can shape group discourse [48]. To mitigate this, moderators were trained to foster inclusive participation, and follow-up emails were sent to all participants to invite additional reflections. Cultural differences between Thailand and the United States also warrant consideration, even though interviews were conducted separately by country. Prior research on communication styles suggested Thais might be more inclined to accommodate audience expectations, whereas Americans might prioritize balancing personal goals with audience interests [49]. These differing cultural norms could have influenced how frontline clinicians and end-line users expressed their views. Furthermore, because interviews with Thai clinicians were conducted in English rather than their primary language, some nuance may have been lost in cross-cultural expression; however, presession proficiency checks, member checking, and transcript review by a bilingual researcher helped mitigate this risk.

While no participants were selected or excluded by demographic or professional criteria, the small sample size remains a limitation. The operation of this mHealth model required 11 frontline clinicians to take dental photos and 5 end-line users to diagnose TDI; all except 1 frontline clinician (a coauthor) participated in this qualitative study. Limited generalizability due to small samples in telemedicine and telehealth research has been noted in an integrated review [50]. Nevertheless, thematic saturation was reached early, with no new codes emerging after the fourth interview, and data collection concluded after 7 sessions. This suggests that the dataset adequately captured the range of user perspectives for this workflow, consistent with Guest et al [51] findings that as few as 6 interviews may be sufficient for thematic analysis and interpretation. Future work should include larger and more culturally diverse clinician samples and explore similar mHealth models to enhance transferability.

The incentive differences between countries stemmed from institutional funding restrictions, which prohibit disbursing school‑supported funds to individuals not legally eligible to work in the United States. Although they did not affect participation rates, such disparities may have introduced subtle response biases or influenced perceived power dynamics. To promote equitable participation in future international research, investigators should consider globally inclusive funding mechanisms or secure domestic support within each participating country.

Interpersonal dynamics [52] and prior researcher-participant familiarity [53] may also influence participant responses. To mitigate potential courtesy bias and power dynamics arising from interviewer familiarity, reflexivity and bracketing strategies were used throughout the study. Moderators received training to foster inclusive dialogue, actively invite dissenting views, and minimize hierarchical influence during interviews. A structured interview guide (Table 1) was used to ensure consistency across sessions and reduce the influence of moderator bias or familiarity on discussion flow. Although the PI observed all sessions, he did not moderate or participate; his webcam and microphone remained off throughout. Participants were not supervised, assessed, or taught by the PI during or after the study period. Their feedback, whether positive or critical, was valued equally and anonymized during reporting to encourage candor. Follow-up emails invited dissenting views, further reducing social pressure and courtesy bias. Prior researcher-participant relationships have also been identified as a potential asset in qualitative research, helping to build rapport and facilitate open dialogue [53].

Future research should incorporate role-specific interview elements, mixed methods approaches, and patient perspectives, alongside cost-effectiveness analyses. With evidence supporting remote assessment of TDI [10], dental caries [11,14], and oral cancer [12], diagnostic capacities could expand to other oral conditions such as tooth wear [54], developmental defects [55], malocclusion [56], and temporomandibular disorders [57].

Scaling mHealth tools in emergency dental settings requires technical and training enhancements as well as policy and infrastructure support. Regulatory frameworks for teledentistry differ across jurisdictions and may lack explicit guidance for photographic diagnosis, raising concerns around licensure, liability, and documentation standards. Reimbursement for asynchronous services—such as image-based consultations—remains inconsistent, potentially limiting uptake in emergency departments and underserved communities. Moreover, integration with existing electronic health record systems and secure data transmission protocols is vital to ensure interoperability, privacy compliance, and operational efficiency. Addressing these regulatory, reimbursement, and infrastructure barriers is essential to move beyond pilot models and establish sustainable, scalable mHealth solutions.

Equally important is the role of educational and professional development in supporting adoption and innovation. Integrating photography-based mHealth workflow into emergency care requires structured training and clear protocols for photo capture and clinical assessment. Competency development in teledentistry should be incorporated into continuing education and dental curricula to prepare both current and future providers. At the system level, health care organizations can leverage the insights from this study to guide infrastructure planning, resource allocation, and policy for effective integration of mHealth technologies.

### Conclusion

Within the study limitations, frontline clinicians and end-line users regarded smartphone-based photography as a feasible, acceptable, and usable mHealth tool for remote dental assessment. Strengthening self‑efficacy through ongoing education and professional development will be essential, while patient and clinician resistance may be mitigated by strategies such as inclusive development, communication, reduction of operational burdens, and targeted training. Technical refinements, including standardized photo templates, integration with patient management systems, and streamlined login, could further enhance usability. Future research should pilot this model in larger and more diverse clinical populations, evaluate cost-effectiveness, and explore its integration into broader oral health workflows beyond TDI.

## Abbreviations

AI: artificial intelligence
COREQ: Consolidated Criteria for Reporting Qualitative Research
CSIRO: Commonwealth Scientific and Industrial Research Organisation
HIPAA: Health Insurance Portability and Accountability Act
mHealth: mobile health
PI: principal investigator
TAM: Technology Acceptance Model
TDI: traumatic dental injury

## Data Availability

The datasets generated or analyzed during this study are not publicly available because participants of this study did not give written consent for their recordings to be publicly shared, but the transcribed data are available from the corresponding author (BH) on reasonable request.
